# Enriching Public Pathogen Genomic Records With Patient Metadata: Quantitative Study and Exploratory Case Analysis

**DOI:** 10.2196/92529

**Published:** 2026-10-06

**Authors:** Michael J Pavia, Karen O'Connor, Graciela Gonzalez-Hernandez, Matthew Scotch

**Affiliations:** 1 Biodesign Center for Environmental Health Engineering, Arizona State University, 1151 S. Forest Ave, Tempe, AZ, United States, 1 855-278-5080; 2 Department of Biostatistics, Epidemiology and Informatics, Perelman School of Medicine, University of Pennsylvania, Philadelphia, PA, United States; 3 Department of Computational Biomedicine, Cedars-Sinai Medical Center, Los Angeles, CA, United States; 4 College of Health Solutions, Arizona State University, Phoenix, AZ, United States

**Keywords:** pathogen genomics, genomic surveillance, metadata standards, bioinformatics, data interoperability

## Abstract

**Background:**

During the COVID-19 pandemic, large-scale sequencing generated millions of SARS-CoV-2 genomes in public repositories including GenBank and GISAID. However, most records lack detailed patient metadata, including demographic information and clinical outcomes. This lack of host-associated information limits their utility for large-scale pathogen genomics analyses. Although sequence records linked to journal publications may contain relevant metadata, systematically extracting and linking this information requires substantial manual effort.

**Objective:**

This study aimed to assess host metadata completeness in GenBank SARS-CoV-2 records and to demonstrate, through an exploratory case study, analytical opportunities enabled by enriched clinical and demographic annotations for genomic epidemiology.

**Methods:**

The authors searched LitCovid for PubMed Central (PMC) articles published between January 2023 and December 2024 that reported original complete SARS-CoV-2 genome sequences deposited in GenBank with sequence-specific patient metadata from human hosts. Two independent reviewers screened eligible articles and manually extracted metadata on sample collection, demographics, treatments, serology, vaccination status, infection presentation, and clinical outcomes. Enriched metadata were defined as patient information in the publication but absent from GenBank records. Synonymous clinical and demographic terms were standardized using SNOMED CT, and Charlson Comorbidity Index scores were calculated when metadata were available. SARS-CoV-2 genomes were retrieved, assembled, and subjected to quality control using standard bioinformatics tools. To demonstrate enriched metadata’s analytical value, we selected a subset of genomes with longitudinal sequencing, comprising 100 genomes from 34 patients across 4 studies for exploratory analysis of within-host viral evolution and patient outcomes.

**Results:**

Among 116,600 articles identified through our PMC/LitCovid screening framework, approximately 0.02% (n=21) reported original complete SARS-CoV-2 genomes deposited in GenBank with accessible sequence-specific metadata. Within this eligible set, GenBank records contained on average 22% completeness for host metadata in our extraction schema. Completeness was confined to sample fields (averaging 81%); host demographic and clinical fields averaged 2%. Manual enrichment increased overall completeness by 30% on average, recovering 56% (14/25) of metadata types absent from GenBank records. In the longitudinal subset, enriched metadata enabled host-stratified analyses, showing nominal associations between immunocompromised status and higher within-host evolutionary rates (*P*=.02) and unique amino acid mutations (*P*=.04). Models with enriched patient and treatment metadata outperformed mutation-only models for mortality, hospitalization, and infection duration.

**Conclusions:**

Enriched host metadata improve the utility of pathogen genomic data by enabling analyses linking viral variation with demographics and clinical outcomes. Despite limited clinical and demographic information in examined GenBank records, manual enrichment facilitated a more comprehensive view of viral evolution and disease dynamics than sequence data alone. The case study’s genotype-phenotype associations are exploratory and require validation in larger, independently collected cohorts. These findings highlight the need for more standardized, structured, and accessible patient metadata deposition with genomic sequences to strengthen pathogen genomics and precision public health research.

## Introduction

The COVID-19 pandemic brought about significant changes in pathogen genomics, with large numbers of viral sequences being shared openly in nucleotide repositories like GenBank [1] and Global Initiative on Sharing All Influenza Data (GISAID) [2]. These databases are essential for answering scientific questions on SARS-CoV-2 or other pathogens, including differences in rates of evolution [3], transmission patterns [4], and geographic variation in the diversity of variants of concern [5]. While these platforms facilitate rapid sharing of genomic data, their utility is limited by inconsistent or incomplete reporting of patient metadata, such as demographics, clinical outcomes, and/or comorbidities [6,7]. This metadata may be described in the accompanying publications, yet sequence records often lack a manuscript reference, either because one does not exist or because the record was never updated to include the publication link [8]. Consequently, the lack of metadata in pathogen genomics limits our capacity to connect viral sequences with patient phenotypes, impeding the identification of key epidemiological patterns.

Integrating metadata with viral genomic data is essential for understanding how viral variation contributes to clinical outcomes. For example, we know that genetic variations in the SARS-CoV-2 spike glycoprotein and host factors, such as angiotensin-converting enzyme 2 (*ACE2*) and transmembrane serine protease 2 (*TMPRSS2*) polymorphisms, directly influence viral infectivity and disease severity [9]. In addition, while comparative analyses across variants have demonstrated that hospitalization rates vary by lineage [10], the associations often lose statistical significance once models account for population-level factors, such as changes in clinical care standards and testing practices [11]. Furthermore, without comorbidity data such as obesity (as measured by BMI), the impact of the A20268G mutation on hospitalization would have been attributed only to viral lineage [12].

Understanding the relationship between viral genotypes and patient clinical outcomes depends on the granularity of available metadata. For instance, Patel et al [13] linked SARS-CoV-2 mutation profiles to age and geography to find that working-age individuals (18‐64 years) carry the highest burden of unique mutations, with high regional variability. Incorporating metadata on vaccination phases further showed distinct spikes in unique mutations that coincided with the vaccine rollout, indicating that demographics, geography, and public health initiatives together influence evolutionary rates. In another example, longitudinal sequencing of SARS-CoV-2 infections in immunosuppressed patients receiving antiviral treatments showed that these individuals harbor a greater number of private (nonlineage) mutations, which would have been missed in coarser datasets [14]. Furthermore, Larsen et al [15] found that the spike protein mutation D614G is linked to shifts in symptom progression (a tendency for cough to precede fever), a relationship that emerged only after the inclusion of detailed clinical symptom data in the analysis. These studies demonstrate that integrating detailed, high-quality patient metadata is critical for clarifying the clinical and public health implications of viral evolutionary dynamics.

Incomplete or ambiguous metadata pose significant challenges for accurate genomic epidemiology. As an example, in a global analysis of SARS-CoV-2 sequences from GISAID, it was observed that 63% of records lacked demographic data and more than 95% were missing patient-level clinical information [16]. Additionally, we have described the lack of host location metadata in pathogen sequence records in GenBank [17,18]. To address this issue, we have developed a framework integrating uncertainty in sampling locations into phylodynamic analysis, rather than relying on fixed geographic assignments [19,20]. This approach outperforms conventional methods when reconstructing viral persistence times, migration rates, and ancestral origins. Additionally, efforts have been made to develop a natural language processing (NLP)–based system designed to automatically extract and refine geospatial data directly from scientific literature [21].

The goal of this study is to demonstrate how sequence-specific host metadata affect the utility of public pathogen sequence data, using SARS-CoV-2 as the primary case study. First, we quantify the availability of patient-level metadata linked to SARS-CoV-2 genomes in GenBank, among recent open-access publications in LitCovid [22]. We then describe the enrichment workflow for extraction, normalization, and linking of patient metadata to sequence records. From this enriched dataset, we use a subset of samples with longitudinal sequencing to demonstrate analyses that are either not possible or are limited when using the metadata stored in GenBank. This case study is intended as exploratory rather than as a definitive test of genotype-phenotype associations. The objective is to illustrate the analytical opportunities enabled by metadata enrichment and to identify candidate associations that warrant evaluation in larger, independently collected datasets. By leveraging metadata-enriched pathogen genomes, we demonstrate an untapped potential for informing more effective responses to emerging viral threats and precision public health research.

## Methods

### Systematic Search, Screening, and Data Enrichment

We searched LitCovid [22] for full-text PubMed Central (PMC) articles published between January 2023 and December 2024. We used regular expressions to screen for (1) mentions of sequence databases (GenBank, BioProject, BioSample, Sequence Read Archive [SRA], and GISAID), (2) strings that matched the alphanumeric GenBank accession number format, and (3) references to variants of interest (VOI) or variants under monitoring (VUM), including BA.2, BA.2.86, JN.1, KP.2, KP.3, KP.3.1.1, LB.1, XEC, JN.1.7, and JN.1.18, as defined by the World Health Organization (WHO) in November 2024. Two reviewers independently assessed articles for all 3 mentions. We specified an inclusion criterion where an article needed to report (1) original SARS-CoV-2 complete genome sequence data, (2) the deposit of the raw reads or consensus sequences in GenBank, and (3) sequence-specific patient metadata derived from human hosts. For this effort, we only considered sequences deposited in GenBank to leverage the National Center for Biotechnology Information (NCBI) *Entrez* environment, which links PMC and GenBank (nucleotide) databases [23]. We therefore excluded articles that submitted data to GISAID or that were derived from nonhuman hosts or from environmental sources such as wastewater.

For articles that met our inclusion criteria, we manually extracted sequence-specific patient metadata encompassing sample collection details, patient demographics, treatment regimens, laboratory results, vaccination status, infection presentation, and clinical outcomes when available. We defined enriched metadata as patient information that was not reported in GenBank. Pre-enriched refers to the metadata that were recovered from GenBank; this metric was different for each article. Metadata that were not reported, missing, or not applicable were recorded as absent without distinguishing between them, and the number of assessed fields remained fixed across studies. Additionally, for each article where enriched metadata contained enough demographic and clinical information, such as age and comorbidities, we calculated the Charlson Comorbidity Index (CCI) [24]; CCI was not calculated when comorbidity data were incomplete. To ensure consistency across studies, we grouped synonymous terms (including sampling location/method, comorbidities, and treatment coding) extracted from articles according to SNOMED CT [25]. Terms that did not map unambiguously were left as initially extracted. A complete breakdown of the LitCovid screening and metadata enrichment protocols can be found in Multimedia Appendices 1 to 3.

We assigned each extracted field to 1 of 2 tiers. Universally expected fields—collection date, country of origin, geographic location, and biospecimen type—describe the sample and apply to any deposited sequence regardless of study design. Context-dependent fields describe demographics, clinical presentation, treatment, and outcome, and their relevance varies based on study design. This distinction was necessary because a missing value carries a different meaning in each tier. Absence of a universally expected field reflects incomplete deposition, whereas absence of a context-dependent field may instead indicate a variable that was not measured or was not applicable.

To evaluate metadata enrichment, we used 4 complementary metrics, each calculated per study and reported as the mean across the 21 studies unless otherwise stated. First, metadata completeness was calculated as the proportion of predefined metadata fields available in GenBank relative to the total number assessed, with missing metadata defined using the same denominator. Second, enrichment completeness was calculated after incorporating metadata extracted from the associated publications. Third, recovered metadata types refer to distinct metadata categories (age, sex, symptoms, hospitalization, mortality, etc) that were absent from GenBank but obtained through manual extraction; the reported percentage represents the proportion of previously unavailable metadata types recovered. Finally, additional metadata variables per sample were calculated as the number of new metadata fields linked to an individual sequence following enrichment. Completeness before and after enrichment was additionally calculated within each tier. Together, these metrics capture changes in overall completeness, recovery of previously unavailable metadata categories, and the number of additional patient-level variables linked to genomic records.

### SARS-CoV-2 Genome Retrieval and Assembly

Using NCBI Entrez Direct [26], we obtained SARS-CoV-2 genomes from GenBank and SRA libraries for 8 of the studies. We assembled the raw Illumina reads by first removing low-quality reads and adapters with fastp [27], followed by IRMA (iterative refinement meta-assembler) for reference-based assembly [28]. For 3 studies where both GISAID-deposited genomes and SRA libraries were available, we used the GISAID genomes solely as an external reference to validate our assemblies, comparing them with BLAST (Basic Local Alignment Search Tool) [29]; GISAID sequences were not used in any downstream analyses. We applied a threshold of >99.5% identity or fewer than 6 combined mismatches and gaps; assemblies that did not meet these criteria were excluded from downstream analyses. We used Nextclade [30] to assign clades, evaluate genome quality, and identify both nucleotide and amino acid mutations relative to the Wuhan-1 genome [NC_045512.2]. Finally, we excluded any genome classified as “bad,” by Nextclade [30] for its overall QC status from downstream analyses.

### Sequence Selection and Metadata Integration for Within-Host Analyses

While genomic data alone can be used to identify amino acid mutations, linking or stratifying these observations with clinical outcomes requires integration with patient metadata. Using the enriched dataset, we studied how the addition of immune status, comorbidities, and treatment regimen metadata improved our ability to understand viral evolution and patient outcomes for SARS-CoV-2 infections. To strengthen generalizability, we combined data from multiple articles in which longitudinal SARS-CoV-2 sequence data were available, as well as corresponding patient metadata encompassing immune status, treatments, and outcomes. This curated dataset consisted of 100 genomes from 34 patients (Figure 1 and Table S1 in Multimedia Appendix 1), drawn from Gonzalez_Reiche_2023 [31], Igari_2024 [32], Manuto_2024 [33], and Pavia_2024 [34].

**Figure 1.**
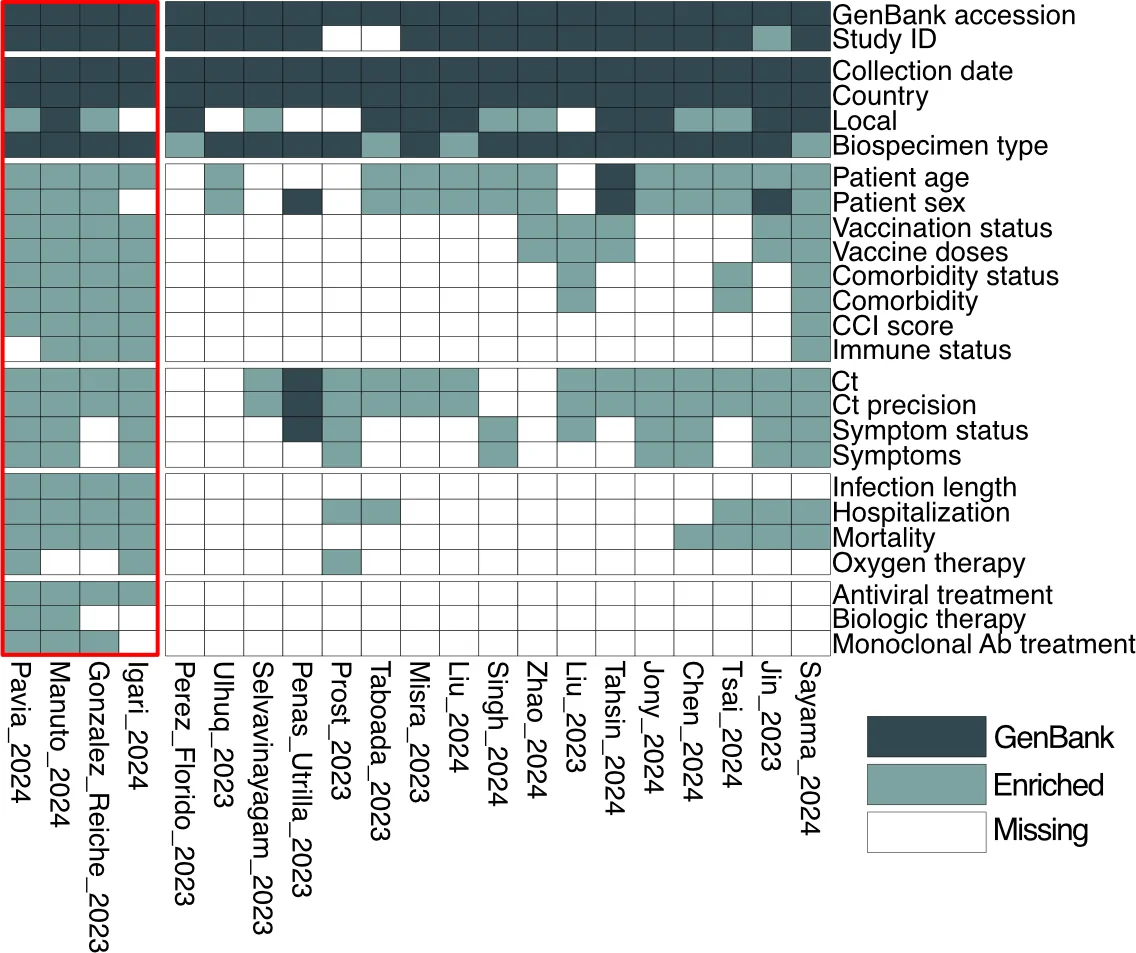
Heatmap of sequence-specific patient metadata collected [31-51]. Dark blocks represent metadata available in GenBank, light blocks represent metadata extracted from publication text, and white blocks represent missing metadata. Fields are grouped into 6 blocks and ordered from study metadata to clinical treatment: study metadata, sample collection, patient characteristics, infection characteristics, disease severity and outcomes, and treatment. Studies highlighted in red were used in the case study. Local refers to state or city vs county level location. Ct refers to the cycle threshold in reverse transcription polymerase chain reaction, with Ct precision indicating either exact value or a reported range. Symptom status refers to whether cases are asymptomatic or symptomatic cases, and symptoms lists the specified reported symptoms. Vaccination status refers to whether a patient was vaccinated, and vaccine dose specifies the number of doses received prior to sequencing. Comorbidity status refers to the presence or absence of comorbidities, and comorbidity lists the specific conditions reported. Ab: antibody; CCI: Charlson Comorbidity Index.

### Case Study: Linking Immune Status to Within-Host Evolution and Outcomes via Enriched Metadata

We analyzed longitudinal SARS-CoV-2 genomes from 4 articles encompassing individuals with varying immune status, comorbidities, and treatment regimens. We performed phylogenetic reconstruction using IQ-TREE (GTR+G model) [52] with 1000 bootstrap replicates. For time calibration, we used the treedater package [53] in R (R Foundation for Statistical Computing) under an uncorrelated clock model. We estimated evolutionary rates for patients with two or more time points by fitting a linear regression of root-to-tip genetic distances against sampling dates and further assessed the dependence of these rates (mutations per site per year per patient) on immune status and viral clade using multiple linear regression. We identified nonrandom recurrent mutations by applying an empirical binomial model to estimate the expected frequencies of amino acid and nucleotide mutations across the cohort. The resulting binomial *P* values were then adjusted using the Benjamini-Hochberg method [54]. We defined empirical recurrence thresholds as the minimum number of patients for which the mutation’s observed frequency yielded a cumulative binomial *P*<.05. Finally, we classified as *recurrent* any mutations that exceeded the threshold and appeared in two or more viral clades. We set recurrence thresholds at a minimum of 11 patients for amino acid mutations and 10 patients for nucleotide mutations (Figure S1 in Multimedia Appendix 1). To assess associations between amino acid mutations, patient risk factors (immune status and CCI), treatment regimens, and outcomes (mortality, hospitalization, and infection duration), we used generalized linear models with logistic regression for binary outcomes (mortality and hospitalization) and linear regression for infection duration. We grouped recurrent amino acid mutations with identical patient-level patterns to prevent redundancy and reduce model complexity. Our models consisted of (1) mutation group only, (2) mutation group plus antiviral treatment, (3) mutation group plus patient factors, and (4) mutation group with antiviral treatment and patient factors. Patient-level covariates and outcomes (immune status, CCI, treatment regimen, hospitalization, mortality, and infection duration) were linked to each genome sequence, such that multiple longitudinal sequences from the same patient shared identical clinical metadata. Models were fit using complete-case analysis; observations with missing values for any predictor or outcome included in a given model were excluded, and no imputation was performed.

To guard against overfitting, given the small cohort, we performed 5-fold cross-validation [55] using caret [56] to report changes in Akaike information criterion (ΔAIC) and Bayesian information criterion (ΔBIC), which we calculated relative to Δ=0, and the Brier score [56] for binary outcomes and root mean square error (RMSE) for infection length. Because the dataset consisted of 100 genomes derived from only 34 patients, with multiple longitudinal samples contributed by some individuals, the folds were not fully independent at the patient level. Consequently, the cross-validation results should be interpreted as measures of internal consistency rather than evidence of external validity or generalizability. The reported performance metrics therefore provide an indication of model robustness within this dataset but do not constitute independent validation of predictive performance. All statistical analyses were carried out in R (version 4.4.2). Reported *P* values are uncorrected for multiple comparisons, and results with *P*<.05 were considered exploratory rather than confirmatory.

### Ethical Considerations

All genomic and clinical metadata used in this study were obtained from publicly available, deidentified sources. No identifiable patient information was collected, accessed, or analyzed in this study. As this study involved only secondary analysis of publicly available, deidentified data, no additional institutional review board approval or informed consent was required.

## Results

### Availability of Patient Metadata in GenBank

A systematic screening of 116,600 recent publications from LitCovid identified a final set of 21 articles that met all inclusion criteria for metadata enrichment (Figure S2 in Multimedia Appendix 1). In 2023, a total of 71,692 publications were listed in LitCovid, of which 68% (n=48,959) of publications were open access and eligible for further analysis. In contrast, in 2024 there were only 44,908 publications with 49% (n=21,790) available as open access. After applying regular expressions to identify GenBank accessions, sequence databases, and SARS-CoV-2 variants, we considered 640 and 442 candidate articles in 2023 and 2024, respectively. Through manual review, we narrowed the list to a total of 21 articles that met all inclusion criteria (10 in 2023 and 11 in 2024) and subjected these to comprehensive metadata collection (Table S1 in Multimedia Appendix 1).

The studies included in our analysis predominantly sampled SARS-CoV-2 in 2022 (16/21, 76%), coinciding with the emergence and global dominance of the Omicron variant and its sublineages [57]. Geographically, most studies were conducted in Asia (13/21, 62%), followed by Europe (6/21, 29%) and the Americas (2/21, 9%). Across the 21 included articles, GenBank completeness was concentrated entirely in the universally expected tier (Figure 1). Sample collection date and country of origin were reported in all 21 studies, and completeness across the 4 fields averaged 81%. Completeness across the 19 context-dependent fields averaged 2%, and 14 of these types, including immune status, comorbidities, treatment regimen, and clinical outcomes, were absent from every GenBank record and were available only in the article text, requiring manual enrichment.

We found a considerable discrepancy between the metadata reported in GenBank records and the metadata available in the text and Multimedia Appendices 1 to 3. Across all studies, there was a wide range of metadata completeness, measured by the number of metadata items extractable from our studies. Pooled across both tiers, GenBank records captured an average of only 22% of host metadata across studies, leaving more than 75% of host metadata missing. Manual enrichment increased metadata completeness by an average of 30% across studies for all metadata that could be extracted. Enrichment acted almost exclusively on the context-dependent tier, raising the average from 2% to 40%, while the universally expected tier changed from 81% to 94%. Additionally, manual enrichment recovered an average of 56% of metadata types absent from the corresponding GenBank records, although the number of recoverable metadata categories varied across studies. Demographic variables such as age (15/21, 71% of articles) and sex (13/21, 62% of articles) were primarily recovered through enrichment. However, disease outcomes (hospitalization, mortality, and symptoms) were reported less frequently: mortality was found in 38% (8/21) of articles, hospitalization in 43% (9/21), and symptoms in 48% (10/21). Reporting was the lowest for infection length (4/21, 19% of articles) and specific treatment regimens, with frequencies for oxygen therapy, antivirals, biologics, and monoclonal antibodies ranging from 9% (2/21) to 19% (4/21).

Some studies, such as Manuto_2024 (37 genomes [33]) and Pavia_2024 (9 genomes [34]), reported nearly all metadata types extractable in our dataset (24/25, 96% complete), representing best practices for data sharing and facilitating downstream analysis and cross-cohort comparisons. In contrast, Peñas_Utrilla_2023 (6 genomes [42]) baseline GenBank metadata were not improved by manual enrichment.

### Evolutionary Dynamics and Risk Predictors in Persistent SARS-CoV-2 Infections

The analyses below are exploratory. They are presented to illustrate what enriched metadata make analytically accessible, not to establish genotype-phenotype associations. Nextclade placed our 100 sequences (from 34 patients across 4 articles) into 5 distinct PANGO (Phylogenetic Assignment of Named Global Outbreak) lineages, led by BA.1 and BA.5, with contributions from Italy, Japan, and the United States (Figure 2A). Apart from collection date, this reflects the analytical limits of the metadata deposited in GenBank for these sequences. Using the enriched metadata available, we could then explore whether within-host evolutionary rates varied as a function of immune status and viral clade using multiple linear regression (Figure 2B). In this exploratory analysis, immunocompromised individuals were significantly associated with higher within-host evolutionary rates (*P*=.02). Among clades, BA.1 (21K) had a near-significant association with higher rates of amino acid mutations (*P*=.052), while the other clades showed no significant differences. We further explored whether the diversity of unique mutations differed between immunocompromised and immunocompetent patients at the nucleotide and amino acid levels, motivated by the effect of immune status on selection, infection duration, and thus the effective evolutionary substitution rate of the pathogen (Figure 2C). While the total number of nucleotide changes did not differ significantly between the 2 groups, immunocompromised individuals did harbor a significantly higher number of unique amino acid mutations (*P*=.04). Overall, we were able to explore the mutational diversity observed across genomes identifying 497 unique nucleotide mutations (299 missense, 143 synonymous, 38 intergenic, and 17 deletions), which resulted in 348 unique amino acid mutations.

**Figure 2.**
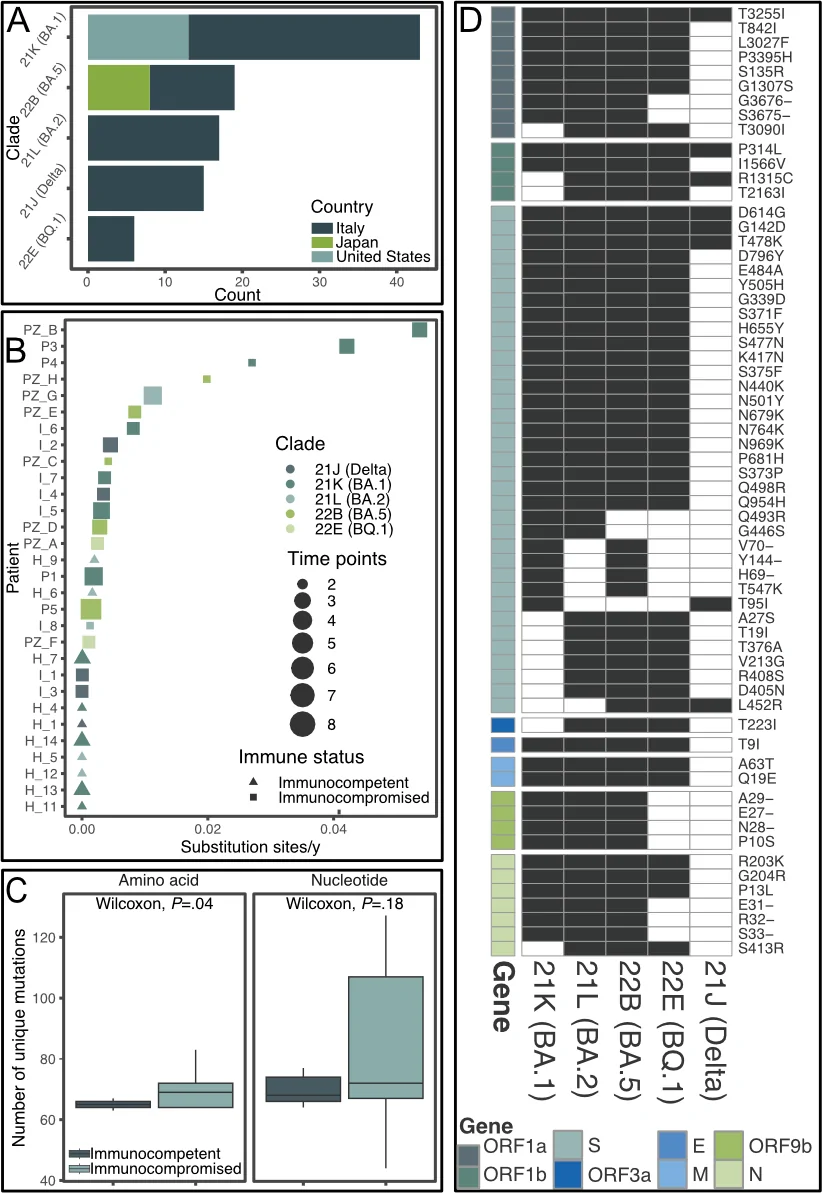
Mutation burden and recurrence across immune states and viral clades. (A) Distribution of SARS-CoV-2 lineages by country. (B) Dot plot of mutation rate per patient. (C) Boxplots comparing the number of unique amino acid and nucleotide mutations between immunocompetent and immunocompromised patients; *P* values from Wilcoxon tests are shown. (D) Heatmap of recurrent amino acid mutations by clade. Black cells represent mutations in each clade (column), white cells represent their absence, and the adjacent color code indicates the corresponding gene for each mutation.

To categorize recurrent mutations from those arising by chance, we performed a rank-frequency analysis and used an empirical binomial model to set the patient-count threshold at which a mutation is unlikely to be a chance event (Figure S1 in Multimedia Appendix 1). Applying this threshold, we identified 63 recurrent amino acid changes and 69 synonymous nucleotide mutations (Figure 2C). Among the recurrent mutations, we identified 2 nucleocapsid mutations R203K+G204R and the Δ31‐33 deletion, both of which are hallmark mutations of the Omicron lineage [58]. Additionally, we identified 2 rare mutations, I1566V (*ORF1b*) and P10S (*ORF9b*), present in less than 0.01% of global sequences [59]. Every recurrent nucleotide mutation corresponded to an amino acid change, except for 2 intergenic mutations, C241T and A28271T. Notably, 35 (55%) of these recurrent amino acid mutations were localized to the spike protein: D405N, E484A, G446S, K417N, L452R, N440K, N501Y, N969K, Q493R, and S371F. While these observations are exploratory and not intended to establish clinical associations, the enriched clinical and genomic metadata provide a framework for the investigation of recurrent mutations and their potential clinical relevance.

We contextualized the amino acid mutations by first grouping them based on congruent patient-level presence and by integrating these groups with patient metadata, including immune status, CCI, and treatment regimens. To evaluate how the addition of clinical and treatment metadata improves the prediction of patient outcomes, we fit a series of nested regression models from mutation-only (equivalent to pre-enrichment analytical capabilities) to those fully adjusted for treatment regimen and patient characteristics (Figure 3). Within each mutation group, we subtracted the lowest AIC (or BIC) value from all models in that group, so the most supported model has Δ=0 and higher values indicate weaker support. Because BIC penalizes each added covariate more heavily than AIC, a model favored by both suggests that the improvement in fit is not explained by model complexity alone. Across 23 (17 were singletons, 74%) groups, the fully adjusted models (including all covariates) demonstrated the strongest performance for ΔAIC and ΔBIC scores in 83% (n=19) of groups for mortality, 100% (n=23) of groups for hospitalization, and 96% (n=22) of groups for infection duration. Cross-validated performance for the fully adjusted models averaged area under the curve (AUC) of 0.93 (Brier=0.09) for mortality, AUC of 0.94 (Brier=0.09) for hospitalization, and RMSE of 19.2 for infection duration (mean across 5-folds). These performance estimates reflect internal evaluation within the cohort and should not be interpreted as evidence that the models will achieve similar performance in independent datasets. External validation in larger and more diverse cohorts will be required to assess generalizability.

**Figure 3.**
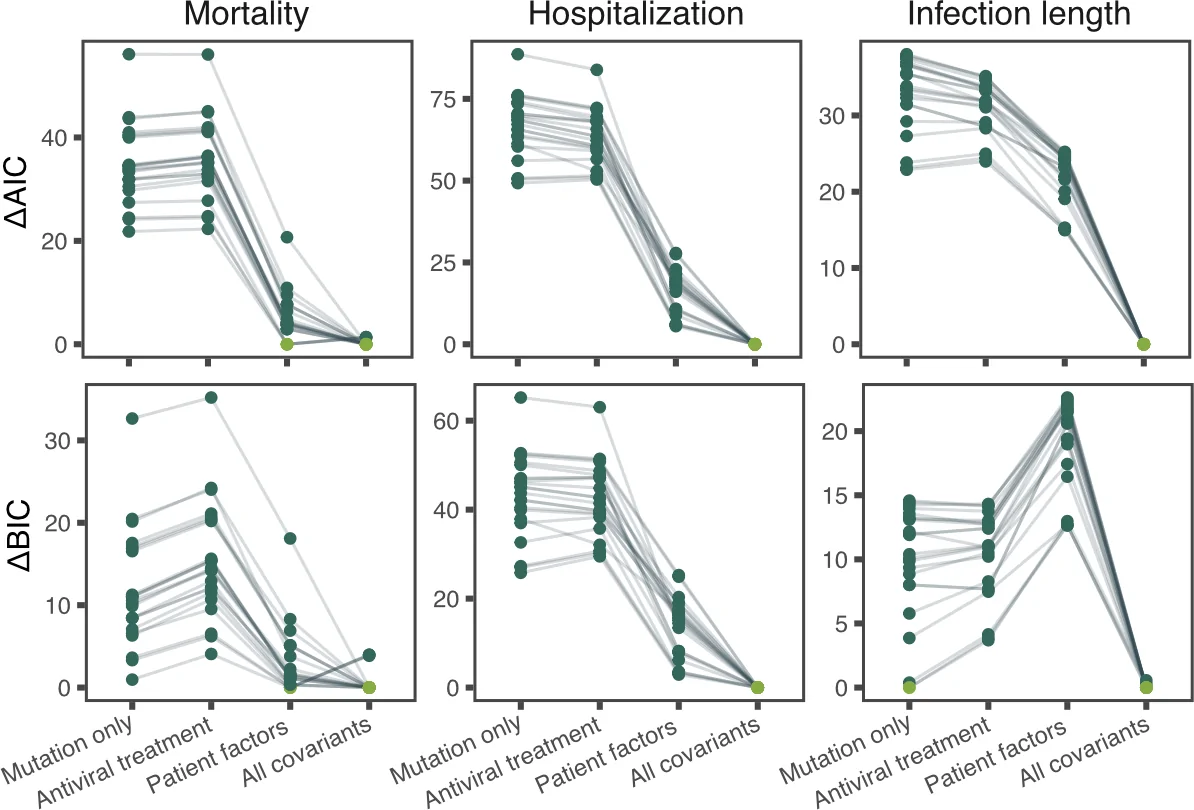
Model comparison for clinical outcomes using changes in Akaike information criterion (ΔAIC) and Bayesian information criterion (ΔBIC). Vertical faceted plots represent the outcome tested by each model. Patient factors include age, Charlson Comorbidity Index, and immune status. Lines link mutation groups across models. The best-fitting model (lowest ΔAIC or ΔBIC) per mutation is highlighted in green.

Using these exploratory models, we identified 2 mutation groups and 10 singletons nominally associated with the tested clinical outcomes (Table 1). Because outcomes were patient-level and the number of events was limited (6 deaths and 13 hospitalizations among 34 patients; infection duration ranged from 2 to 86 days; Table S2 in Multimedia Appendix 1), all genotype-phenotype analyses should be interpreted as hypothesis-generating rather than as estimates of effect. For mortality, 2 groups were nominally associated: a large cluster spanning structural genes (mutations in *S* and *N*) and nonstructural genes (mutations in *ORF1ab* and *ORF3a*). The second group showing nominal association with mortality was a collection of 4 mutations, all occurring within *ORF1a*. Amino acid mutations nominally associated with hospitalization were primarily localized to the *S* gene, within the receptor-binding domain and upstream of the polybasic furin cleavage site. For infection length, only 2 mutations (G446S and T547K) were nominally associated, both within the spike protein.

**Table 1.** Mutation groups nominally associated with clinical outcomes in the fully adjusted model (n=100)^a^.

Outcome, group, and gene	Mutation	Effect size (95% CI)	Direction	*P* value
Mortality^b^				
Group 1		6.28 (1.16‐50.2)	Higher	.03
* N*	S431-			
* ORF1a*	T3090I			
* ORF1b*	T2163I			
* ORF3a*	T223I			
* S*	T19I, V213G, T376A, D405N, and R408S			
Group 2		7.69 (1.49‐61.0)	Higher	.01
* ORF1a*	S135R, T842I, G1307, and L3027F			
Singleton				
*ORF1a*	T3255I	0.05 (0-0.43)	Lower	.01
* ORF1b*	R1315C	6.28 (1.16‐50.2)	Higher	.03
* S*	Y144-	0.14 (0.02-0.71)	Lower	.01
Hospitalization^b^				
Singleton				
* S*	S477N	40.4 (5.74‐510)	Higher	<.001
* S*	E484A	5.56 (1.09‐40.2)	Higher	.04
* S*	P681H	8.19 (1.62‐57.7)	Higher	.01
* ORF1a*	P3395H	40.4 (5.74‐510)	Higher	<.001
* ORF1b*	I1566V	16.9 (3.08‐137)	Higher	<.001
Infection length^c^				
Singleton				
* S*	T547K	15.4 (6.28‐24.5)	Longer	.001
* S*	G446S	11.7 (2.39‐21)	Longer	.02

a Groups were retained if the mutation group had an uncorrected *P*<.05. Logistic regression was used for mortality and hospitalization, and linear regression for infection duration.

b The outcome was assessed using odds ratios as the effect measure.

c The outcome was assessed using β, expressed in days, as the effect measure.

## Discussion

### Principal Findings

In this study, we demonstrate that systematically enriching pathogen genomic records with sequence-specific patient metadata expands the analytical scope of downstream epidemiological investigations. Of the 116,000 articles captured by our screen in 2023 to 2024, approximately 0.02% provided readily accessible sequence-specific patient metadata. This highlights the limitations faced by scientists performing secondary data analysis, as well as deficiencies in data deposition standards, corroborating earlier reports of gaps in genomic data stewardship [7,60]. Through manual curation, we recovered a median of 14 additional metadata variables per sample, including age, sex, comorbidities, treatment, and clinical outcomes, increasing overall metadata completeness by an average of 30%. This enriched dataset supported 2 exploratory analyses that the GenBank records alone could not support: within-host evolutionary rate as a function of immune status, and genotype-phenotype association models incorporating clinical covariates. Collectively, we illustrate how richer host information can significantly increase the amount of usable information in sequence databases for precision public health inquiry.

The volume of metadata stored exclusively in unstructured formats, such as text, figures, and supplementary materials, presents significant challenges for scalability and data reuse. Although our manual extraction efforts partially address this gap, the approach is labor-intensive and impractical as the number of articles increases. Demographic data, such as age and sex, were the most commonly recovered enriched metadata types (relative to pre-enriched metadata), reflecting standardized sampling strategies and their importance in epidemiologic study design [61]. In contrast, clinical information was rarely recoverable and, when present, was found as unstructured text or embedded in figures. This scarcity is further compounded by inconsistencies in clinical documentation and by privacy regulations enforced under the Health Insurance Portability and Accountability Act or the General Data Protection Regulation.

Addressing this gap will require both policy and technical interventions, such as enforcement of metadata deposition standards, data sharing frameworks that provide as much metadata as possible without sacrificing patient confidentiality, and NLP-based tools for metadata extraction. Traditional clinical NLP has been used to map narrative text to standardized codes with expert-level accuracy, accounting for modifiers, including negation, temporal information, and family history [62]. More recently, machine learning pipelines have been developed for the large-scale detection of patient metadata from COVID-19 literature [63]. Incomplete or ambiguous geospatial metadata pose significant challenges for accurate genomic epidemiology [17,18]. To address this issue, frameworks have been developed that integrate uncertainty in sampling locations into phylodynamic analysis [19,20]. Additionally, NLP-based systems have been developed to automatically extract and refine geospatial data directly from scientific literature [21]. Klein et al [63] show that fine-tuned models pretrained on biomedical corpora, such as BiomedBERT, outperform large language models for classifying text-containing metadata. Until NLP-based tools are developed and adopted, demographic data will remain more accessible than clinical metadata, limiting population-level efforts to identify epidemiological trends.

In our case study, genomes recovered from immunocompromised patients contained more amino acid mutations than those from immunocompetent patients, though the small cohort of 34 patients limits the extent to which this comparison can be generalized. Although lineage BA.1 showed a borderline association with elevated rates, we found that, in our small cohort, overall viral clades had minimal impact on evolutionary dynamics compared to host immune status. Infections in severely immunosuppressed individuals, such as those with hematologic malignancies or organ transplants, can persist for months, effectively allowing the virus to undergo continuous adaptation under reduced immune pressure [64]. This extended evolutionary window not only increases the total number of mutations but also shifts the selective pressures acting on the viral population. We observed that immunocompromised patients harbored significantly more unique amino acid mutations, while unique nucleotide changes showed no significant difference between groups. The higher rates of nonsynonymous mutations may suggest that this pattern is consistent with positive selection rather than neutral drift alone influencing the viral population [65], but we did not test this selection directly.

We have shown the utility of integrating detailed clinical metadata with pathogen genomic data for exploratory evaluation of mutational dynamics and clinical outcomes. Specifically, we identified 63 mutations that had a low probability of occurring by chance and recurred across multiple patients and viral lineages. Of these, the majority were localized to the spike protein, including many known to be associated with antibody escape (D405N, E484A, G446S, K417N, L452R, N440K, N501Y, N969K, Q493R, and S371F) [66-71]. These patterns of convergent mutations are comparable to those noted in other studies of persistent SARS-CoV-2 infections in immunocompromised hosts [72]. Separately, we also recovered 2 intergenic nucleotide mutations (C241T and A28271T), whose recurrence might suggest some potential regulatory functions that warrant further investigation.

By leveraging the increased analytical resolution provided by enriched clinical covariates, we identified exploratory associations between mortality and a pattern of mutations that spans both structural and nonstructural genes. Specifically, mutations in the *S* gene (T19I, V213G, T376A, D405N, and R408S) have been shown to enhance the virus’s ability to bind and enter host cells [66,67]. The nonstructural mutations (T3090I, T2163I, and R1315C) are found in genes known to be involved in both disabling host innate immune signaling and controlling the kinetics of viral replication [73]. These functional roles were established in experimental systems other than ours and describe why the mutations are plausible candidates; they are not evidence that the mutations influenced mortality in this cohort. Together, these mutations co-occurred with mortality in our models and may reflect combined viral and host factors (age and comorbidities) that delay or prevent the immune response, although this association will need to be explored further.

Most of the mutations we identified as being associated with hospitalization were localized to the binding domain of the spike gene. Mutation S477N is common among Omicron variants and is known to increase ACE2 affinity, suggesting that one possible explanation is enhanced viral entry, as reported in prior studies [74]. The mutations associated with infection length, G446S and T547K, have been linked to altered T-cell recognition [75] and viral fusion phenotypes [76], respectively. While more efficient viral entry has been associated with more severe symptoms, SARS-CoV-2 persistence was associated with mutations previously linked to immune status [72]. Together, these observations illustrate the potential of integrated, metadata-enriched pathogen genomics in facilitating analysis that can lead to genotype-phenotype relationships with larger cohorts.

This study illustrates the significant, yet often unrecognized, advantage of enriching sequence databases with demographic and clinical metadata. Recent audits have shown that approximately 60% of GISAID submissions are missing data [59] or contain errors or ambiguities [7]. Combined, these lead to misguided genotype-phenotype analyses, compromising the accuracy and reliability of epidemiological studies. Adopting structured and harmonized contextual data standards, such as those developed by the Public Health Alliance for Genomic Epidemiology, would mitigate these issues and maximize the utility of sequencing data across databases and institutions [77]. These standards separate fields by requirement level, and our results indicate where each level matters. The universally expected fields are already nearly complete in GenBank and could be enforced upon required at deposition. The larger deficit lies in the context-dependent clinical fields, whose deposition is constrained both by their relevance to a given study and by patient confidentiality.

### Limitations

Our study has several limitations that should be considered when interpreting our findings. First, our literature search was restricted to open-access articles published in 2023 and 2024 with genomic data available in GenBank; this approach omits relevant articles behind paywalls and excludes genomic data that are only available in other databases such as GISAID. Additionally, our screening strategy required mentions of designated VOIs or VUMs, as defined by the WHO, to identify candidate articles for manual review. While this approach improved specificity and reduced the number of articles that required full-text assessment, it may have excluded otherwise eligible studies. Consequently, our estimate of metadata availability should be interpreted as representative of the literature captured by this screening strategy rather than an exhaustive assessment of all SARS-CoV-2 sequencing studies.

Our approach led to a dataset that was both geographically and temporally skewed toward studies conducted in Asia (13/21, 62%) and in 2022 (16/21, 76%), which limits the broader applicability of our findings to other regions, populations, and phases of the pandemic. Furthermore, despite combining data across 4 articles, our analysis included only 34 patients. We did not adjust for study source, calendar period, or geographic origin because they were correlated with immune status and treatment availability across the 4 studies. In addition, many mutation groups were rare, with 74% (17/23) observed in only a single patient. As a result, coefficient estimates may be unstable, and intervals should be interpreted cautiously. Although 5-fold cross-validation was used to evaluate internal model consistency, it does not provide independent validation and may overestimate predictive performance in small datasets. Finally, while manual enrichment was effective, the labor-intensive process may introduce inconsistencies in metadata collection, reinforcing the critical need for standardized, structured, and accessible deposition of patient metadata alongside genomic sequences.

### Conclusions

Here, we show the importance of enriched host metadata in understanding both viral evolution and clinical dynamics. Linking pathogen genomic data with patient characteristics provides a more comprehensive picture of disease behavior. There have been few large-scale SARS-CoV-2 sequencing studies that have incorporated rich patient metadata, largely because most publicly available genomic sequences contain limited clinical information. Our research directly quantifies this problem and shows how improvements in metadata availability strengthen pathogen genomics. Incorporating enriched metadata has facilitated the identification of genotypic and phenotypic patterns that sequence data alone cannot demonstrate. Comprehensive patient metadata can provide insights into the interplay among viral mutations and clinical outcomes, offering a more complete understanding of viral evolutionary behavior within populations.

## Supplementary material

10.2196/92529 Multimedia Appendix 1 Rank frequency and empirical threshold for nonrandom mutations (Figure S1); number of article records retained at each stage of the filtering process (Figure S2); characteristics of the 21 SARS-CoV-2 sequencing studies included in metadata enrichment (Table S1); and characteristics of the 21 SARS-CoV-2 sequencing studies included in metadata enrichment (Table S2).

10.2196/92529 Multimedia Appendix 2 Supplementary methods detailing the screening protocol used to select the 21 articles and the enrichment procedures applied to extract and normalize patient metadata.

10.2196/92529 Multimedia Appendix 3 Enriched dataset of patient metadata linked to each GenBank accession.

## Abbreviations

*ACE2*: angiotensin-converting enzyme 2
AIC: Akaike information criterion
AUC: area under the curve
BIC: Bayesian information criterion
BLAST: Basic Local Alignment Search Tool
CCI: Charlson Comorbidity Index
GISAID: Global Initiative on Sharing All Influenza Data
IRMA: iterative refinement meta-assembler
NCBI: National Center for Biotechnology Information
NLP: natural language processing
PANGO: Phylogenetic Assignment of Named Global Outbreak
PMC: PubMed Central
RMSE: root mean square error
SRA: Sequence Read Archive
*TMPRSS2*: transmembrane serine protease 2
VOI: variants of interest
VUM: variants under monitoring
WHO: World Health Organization
