# Supplementary material for: Enriching Public Pathogen Genomic Records With Patient Metadata: Quantitative Study and Exploratory Case Analysis
Source: JMIR Bioinform Biotechnol. 2026 Oct 6;7:e92529. doi: 10.2196/92529 (PMC13641315; doi:10.2196/92529)
Supplement: Supplementary file 2 [file bioinform-v7-e92529-s002.pdf]

Supplementary Methods

The screening and enrichment workflow consisted of two stages (1) a hybrid automated/manual screen of LitCovid corpus to identify articles that simultaneously reported original SARS-CoV-2 genomic data and sequence-specific patient metadata, and (2) a fully manual text extraction enrichment in which patient-level metadata absent from GenBank were extracted from the eligible articles and normalized to a controlled vocabulary. Both stages were performed by two human reviewers operating independently.

Screening Protocol

LitCovid was queried for all PubMed Central (PMC) full-text records with a publication date between 1 January 2023 and 31 December 2024. The query returned 116,600 records (71,692 in 2023; 44,908 in 2024). Records were retained only if the corresponding PMC manuscript was open access (at the time of access) reducing the working corpus to 48,959 articles in 2023 and 21,790 articles in 2024, for a combined 70,749 candidates carried forward into the automated screen.

The full text and supplementary material of each candidate article were searched with three categories of regular expressions, applied in the following order:

1. Sequence repository mentions using case insensitive substring matches against the controlled list (GenBank, BioProject, BioSample, SRA, GISAID)
2. Alphanumeric GenBank accession strings
3. SARS-CoV-2 variants using string matches against the controlled list (BA.2, BA.2.86, JN.1, JN.1.7, JN.1.18, KP.2, KP.3, KP.3.1.1, LB.1, XEC).

The code used for regular expression article screening and identification of candidate publications is publicly available at DOI: https://doi.org/10.5281/zenodo.17595095. If an article met at least one of the above criteria they were assessed in the next step. Articles passing this stage totaled 1,082 (640 in 2023; 442 in 2024). Two reviewers, working independently against three binary inclusion criteria. Reviewers were not blinded to authorship, journal, or institution. The criteria were applied in the order listed and failure on any single criteria resulted in exclusion.

1. The article reported newly generated SARS-CoV-2 genome sequences (or the raw reads) produced by the study authors. Review articles, meta-analyses, or reanalysis of existing public assemblies, gene only studies, and computational simulations were excluded.
2. Raw reads and/or consensus assemblies from criterion 1 were deposited in GenBank (or its associated SRA/BioProject/BioSample sub-databases). Articles depositing exclusively to GISAID or other repositories were excluded.
3. For each individual GenBank deposited sequence the article had to permit the unambiguous assignment of at least one piece of patient metadata. This required either a table with corresponding accessions, figure panel with patient metadata, or a

supplementary file in which the host-level information could be traced back to a specific accession. Statements that could not be resolved to individual sequences were treated as not meeting this criterion. Sequences derived from non-human hosts (animal reservoirs, environmental samples, or wastewater) were excluded.

No articles were excluded based on language, sample size, study design, or perceived study quality. The screen was strictly a measure of eligibility against the four criteria above. The screen yielded a final cohort of 21 articles (10 from 2023 and 11 from 2024).

Enrichment Procedures

Enrichment was designed to recover, for each of the 21 included articles, the largest set of sequence-specific patient metadata fields that could be unambiguously linked to a deposited GenBank accession but were absent from the corresponding GenBank record. The enrichment was performed entirely by manual review of the published literature by the same two reviewers who carried out the eligibility screen. Pre-enriched metadata refers to the fields populated within the GenBank record itself at the time of access. Enriched metadata refers to any additional patient-level field that could be assigned to one or more specific GenBank accessions through manual review of the corresponding article and its supplementary materials.

A controlled set of fields were extracted whenever the article supported their assignment to a specific accession: sample collection date, country of origin, state/province or city, specimen sampling site (e.g., nasopharyngeal swab, saliva), RT-PCR cycle threshold value and the precision of its reporting (exact value vs. range), patient age, patient sex, immune status (immunocompromised vs immunocompetent), vaccination status and number of doses received prior to sampling, comorbidities (unstructured list), comorbidity status (binary, comorbidities listed or not listed), symptom status (asymptomatic vs. symptomatic) and reported symptoms (unstructured list), infection length, treatment regimen (antivirals, monoclonal antibodies, biologics, and oxygen therapy), hospitalization, and mortality. Immune status was coded solely on article text (or coding) of the patient; immunocompetency was never inferred. Antivirals included remdesivir and nirmatrelvir-ritonavir; monoclonal antibodies included immunoglobulin, bebtelovimab, casirivimab, imdevimab, bamlanivimab, etesevimab, tixagevimab, cilgavimab, and sotrovimab; biological therapy included, Anti-CD20 mAbs with or without intravenous immunoglobulin.

For each of the 21 included articles, the two reviewers extracted the target fields by reading the full text, all figures, all tables, and all supplementary files. Three extraction modes were used in the following order of priority:

1. Where the article provided a table or supplementary spreadsheet that explicitly mapped GenBank accessions (or unique patient identifiers that resolved to GenBank accessions) to demographic, clinical, sampling, or treatment fields, values were transcribed verbatim

2. Sample collection method, sampling site, country of origin, and sub-national location, when not given on a per-patient basis but described as uniform across the cohort in the Methods section (or inferred from elsewhere in the paper), were assigned identically to every accession from that article.
3. When an article defined its cohort by an explicit clinical condition (e.g., "lung transplant recipients", "patients hospitalized with severe COVID-19"), the corresponding comorbidity, immune status, or hospitalization status was assigned to all accessions from that cohort.

Sampling site, sampling method, comorbidity, and treatment terms were mapped to SNOMED CT concepts. Terms that did not map unambiguously to a single concept were retained as originally reported. No back-translation, additional ontology, or controlled vocabulary was applied to the remaining fields, which were stored as extracted.

The complete enriched dataset is provided as Supplementary_Data.csv and is dynamically accessible at https://dataviz.hlpgonzalezlab.com/genomic-metadata.
